# Enhancing Virion Tethering by BST2 Sensitizes Productively and Latently HIV-infected T cells to ADCC Mediated by Broadly Neutralizing Antibodies

**DOI:** 10.1038/srep37225

**Published:** 2016-11-17

**Authors:** Tram N. Q. Pham, Sabelo Lukhele, Frédéric Dallaire, Gabrielle Perron, Éric A. Cohen

**Affiliations:** 1 Institut de Recherches Cliniques de Montréal, Montréal, Québec, H2W 1R7, Canada; 2 Department of Microbiology, Infectiology and Immunology, Université de Montréal, Montréal, Québec, H3T 1J4, Canada

## Abstract

Binding of anti-HIV antibodies (Abs) to envelope (Env) glycoproteins on infected cells can mark them for elimination via antibody-dependent cell-mediated cytotoxicity (ADCC). BST2, a type I interferon (IFN)-stimulated restriction factor that anchors nascent Env-containing virions at the surface of infected cells has been shown to enhance ADCC functions. In a comprehensive analysis of ADCC potency by neutralizing anti-HIV Abs (NAbs), we show in this study that NAbs are capable of mediating ADCC against HIV-infected T cells with 3BNC117, PGT126 and PG9 being most efficient. We demonstrate that HIV-induced BST2 antagonism effectively attenuates Ab binding and ADCC responses mediated by all classes of NAbs that were tested. Interestingly, IFNα treatment can reverse this effect in a BST2-dependent manner. Importantly, while reactivated latent T cell lines display some susceptibility to ADCC mediated by broadly NAbs, inactivating BST2 viral countermeasures and/or exogenous IFNα augment their elimination. Overall, our findings support the notion that NAbs can induce ADCC. They highlight that while BST2 antagonism by HIV promotes ADCC evasion, strategies aimed at restoring BST2 restriction could improve anti-HIV responses and potentially provide a means to eliminate reactivated cells in latent reservoirs.

Human immunodeficiency virus (HIV)-type 1 enters target cells, primarily CD4^+^ T cells and macrophages, through sequential interactions between viral envelope (Env), composed of a trimer of gp120 and gp41 heterodimers, and cell surface receptors CD4 and CCR5 (or CXCR4)^1^. Each interaction causes conformational changes in Env, and in turn enable a subsequent phase of the entry process. Binding of gp120 to receptor CD4 causes the trimer to assume a structure (CD4-induced or CD4i) that allows gp120 to bind co-receptor CCR5 or CXCR4. Co-receptor engagement triggers additional remodeling within the gp41 transmembrane subunits, rearranging them into a stable six-helix bundle that facilitates fusion between viral and cellular membranes. This multi-stage mechanism of entry allows HIV-1 to mask conserved functional sites from humoral immunity^2^,^3^.

HIV infection triggers production of antibodies (Abs) against Env gp120 and gp41 subunits, some of which can bind free virus and prevent new infection. While Abs capable of neutralizing the infecting virus maybe readily produced, only 20–30% of patients make Abs that can neutralize a broad spectrum of viruses, and typically after several years^4^,^5^,^6^,^7^. These so-called broadly neutralizing Abs (bNAbs) target the CD4-binding site (CD4-bs) on gp120, glycans on the V1/V2 apex of gp120, V3-glycans on gp120, the membrane proximal external region (MPER) on gp41 as well as the gp120-gp41 interface. Passive transfers of bNAbs have been shown to protect macaques and humanized mice from challenges with simian-HIVs or HIV-1, respectively^8^,^9^,^10^,^11^, and to interfere with establishment of reservoirs in humanized mice^12^. In human studies, a single infusion of CD4-bs Ab 3BNC117 reduced viral load by up to 2.5 log^13^. Although it was implied that the protective effects of Abs required Fc - Fc receptor engagement^8^,^9^,^12^,^14^,^15^, the involvement of antibody-dependent cell-mediated cytotoxicity (ADCC) was only directly addressed in some studies^12^,^14^.

HIV-1 infection downregulates CD4^16^ and BST2^17^,^18^ from the surface of infected cells and such modulation correlates with reduced ADCC activity^19^,^20^,^21^,^22^. BST2 is a type I interferon (IFN-I)-upregulated restriction factor that tethers nascent virions at the surface of infected cells, thereby preventing their efficient release^17^,^18^. HIV-1 Vpu-mediated antagonism of BST2^17^,^18^ conceivably leads to reduced levels of tethered Env-containing virions and less efficient recognition of infected cells by ADCC-mediating Abs. In addition, decreasing CD4 expression by Nef and Vpu^16^ presumably prevents Env from engaging CD4, a step that is necessary to uncover certain CD4i, ADCC-promoting epitopes on Env. An example of such epitopes is that recognized by the non-neutralizing A32 Abs. It is currently not understood whether different classes of bNAbs are subjected to ADCC evasion by Nef and Vpu. Nor is it entirely defined whether reactivated HIV latent cells are susceptible to ADCC^23^,^24^, and if modulating activities of Nef and Vpu would alter susceptibility of latent cells to ADCC by bNAbs.

Here, we surveyed a panel of anti-Env Abs, that target all known vulnerable regions of Env, for their ability to mount ADCC response against infected T cells. We show that bNAbs mediate ADCC with varying efficiencies. We further demonstrate that Vpu and Nef differentially modulate ADCC activities. Moreover, inactivating BST2 antagonism by HIV-1 enhances Env recognition and, consequently, ADCC activities mediated by all classes of NAbs. Similarly, exogenous IFNα treatment heightens ADCC response against productively infected CD4^+^ T cells in a BST2-dependent manner. Lastly, we reveal that this approach could sensitize reactivated latent cells to ADCC. Overall, our study suggests that strategies aimed at improving ADCC function using IFNα and/or small molecule inhibitors of BST2 antagonists represent a promising avenue to promote a more effective elimination of productively infected cells and clearance of latent viral reservoirs in “Shock and Kill” HIV cure approaches.

## Results

### Neutralizing Abs mediate efficient ADCC against CEM CD4^+^ T cells infected with different HIV-1 primary isolates

**Figure 1:**
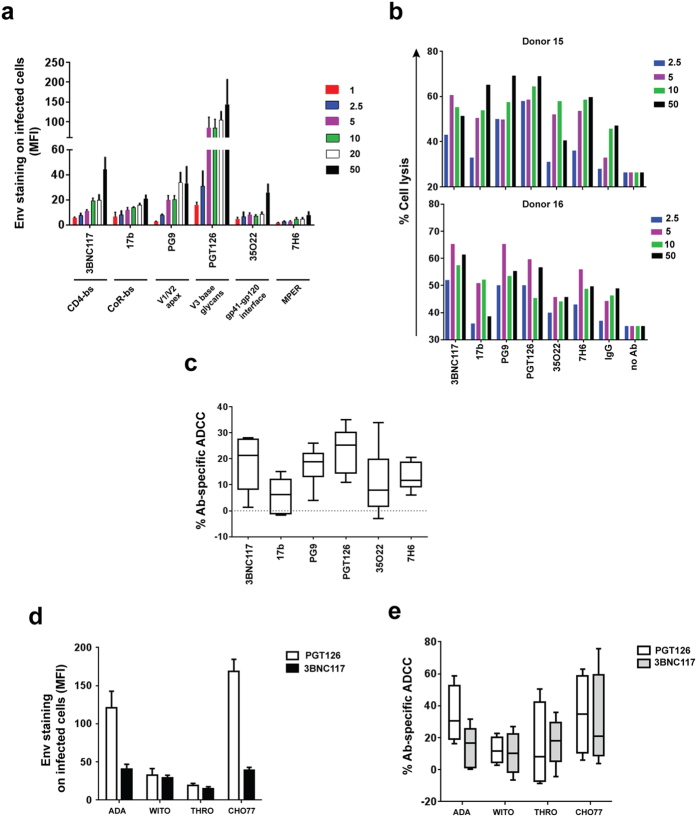
Neutralizing Abs can mediate efficient ADCC against T cells infected with different HIV isolates. (**a**–**e**) CEM CD4^+^ T cells were infected with prototypic CCR5-tropic NL4.3.ADA.IRES.GFP wild-type (WT) or (**d** and **e**) the indicated T/F viruses and analyzed by flow cytometry for Env expression (**a** and **d**) and susceptibility to bioluminescence-based ADCC (**b**, **c** and **e**) using indicated anti-HIV NAbs (in μg/ml). (**a**) Extent of Env staining (in median fluorescence intensity (MFI) units) on GFP^+^ T cells (n = 3 to 4). (**b**, **c** and **e**) PBMCs from healthy donors were used as effector cells. Percentages of Ab-specific ADCC were determined following subtraction of cell lysis mediated by control IgG from that by test antibody (5 μg/ml). (**b**) ADCC responses by two donors as examples and (**c**) Compilation of median ADCC activity from at least 8 different donors. (**d**) Median Env staining on p24^+^ T cells from 3 experiments. (**e**) Boxes and whiskers graph showing median ADCC response from 4 donors. MPER, membrane proximal external region.

First, we examined how efficient neutralizing Abs recognize their epitopes on T cells infected with prototypic wild-type HIV-1 expressing the ADA Env. While most Abs in the series were broadly neutralizing, 17b (CoR-bs) was included in the analysis to have representative Abs recognizing all known regions on Env. Under our experimental conditions (staining performed at 4 °C), the Abs bound Env with varying efficiencies and, unsurprisingly, saturated at different concentrations (2.5 to 10 μg/ml). At a given dose, Abs which target the N332-V3 glycan (PGT126) bound most strongly (several fold higher) than the others (Fig. 1a). In contrast, Abs which recognize the MPER (7H6) or gp120-gp41 interface (35O22) bound least strongly on infected cells, even at the highest concentration tested. Screening of additional Abs showed similar results (see Supplementary Fig. S1). At near saturation concentration (5 μg/ml), PGT126 was most potent at mediating ADCC, followed by PG9 (targeting V1/V2 glycans) and 3BNC117 (Fig. 1b and c). For 35O22 and 17b, which showed minimal ADCC activity at 2.5 μg/ml (see Supplementary Fig. S1), increasing Ab concentration to 10 μg/ml improved killing of infected cells (Fig. 1c; see also Supplementary Fig. S1). Nevertheless, for more potent Abs (i.e., PGT126, PG9 and 3BNC117), such increases were not observed, presumably because of saturation. Although 7H6 recognized the ADA Env most weakly, its median ADCC activity was higher than those of 17b and 35O22, both of which bound Env more efficiently than 7H6. Such a lack of correlation between Env binding and ADCC response could be due to differential stabilities of Ab-Env complexes at 37 °C (ADCC assay) versus at 4 °C (Env binding assay), as has also been observed by others^24^. Additionally, at an exceedingly high concentration of 50 μg/ml of antibodies, although there was an increase in Env recognition for a couple of antibodies, this did not necessarily translate to more efficient antibody-specific ADCC (Fig. 1a and 1b, see also Supplementary Fig. S1).

In assessing if ADCC activity was correlated with neutralization capability, we found that ADCC-potent PGT126, PG9 and 3BNC117 were also effective at neutralization, with IC_50_ ranging from 0.04 to 0.07 μg/ml (see Supplementary Fig. S1). Likewise, 7H6, 17b and 35O22 which were less capable of mediating ADCC were also not as potent at neutralizing (IC_50_: 0.26, 1.3 and 7.5 μg/ml, respectively). The results with 17b, and especially 35O22, likely reflect poor Ab binding when Env is in a closed, unliganded conformation^6^,^25^, at least in the context of this virus.

Based on findings with the prototypic virus, PGT126 and 3BNC117 were selected for further analyses with CEM CD4^+^ T cells infected with transmitted/founder (T/F) viruses. First, PGT126 recognized its epitope much more efficiently than 3BNC117 on CHO77 virus-infected T cells. However, epitope recognition on T cells infected with WITO or THRO isolates was comparable between the two Abs, albeit markedly lower than that of the other two viruses (Fig. 1d). It is less likely that these observations were due to unequal levels or extents of infection since the proportion of p24^+^ cells was similar among the different strains, and the observations were independent of the intensity of p24 staining per cell. Importantly, the extent of ADCC activity largely mirrored that of Env recognition except for THRO virus where despite minimal binding, 3BNC117 could still induce significant cell lysis (Fig. 1e). Overall, our data indicate that NAbs can trigger elimination of infected T cells expressing Env proteins from different primary virus isolates. The variable ADCC responses between virus isolates might be related to surface stability of NAbs and/or differential exposure of relevant epitopes.

### HIV-1 accessory proteins Vpu and Nef modulate Env recognition by NAbs on infected T cells

The variation in the ability of the same antibody to recognize Env, hence mediate ADCC in T cells infected with different isolates points to differential capabilities of these viruses to shield infected cells. Given previous findings demonstrating that HIV exploits Nef and Vpu to modulate Env recognition and evade ADCC^19^,^20^,^21^,^22^, CEM CD4^+^ T cells were infected with prototypic WT strain or its variants lacking Vpu (U-, ΔU), Nef (N-, ΔN) or both proteins (N-U-, ΔNΔU). The ΔNΔU D368A virus was included in the analysis to delineate the importance of CD4-Env engagement in the recognition of target cells by NAbs. Substitutions at residue 368 within the CD4-bs of Env disrupt CD4-Env interactions^26^. To this end, we observed several Env recognition profiles with these viruses.

**Figure 2:**
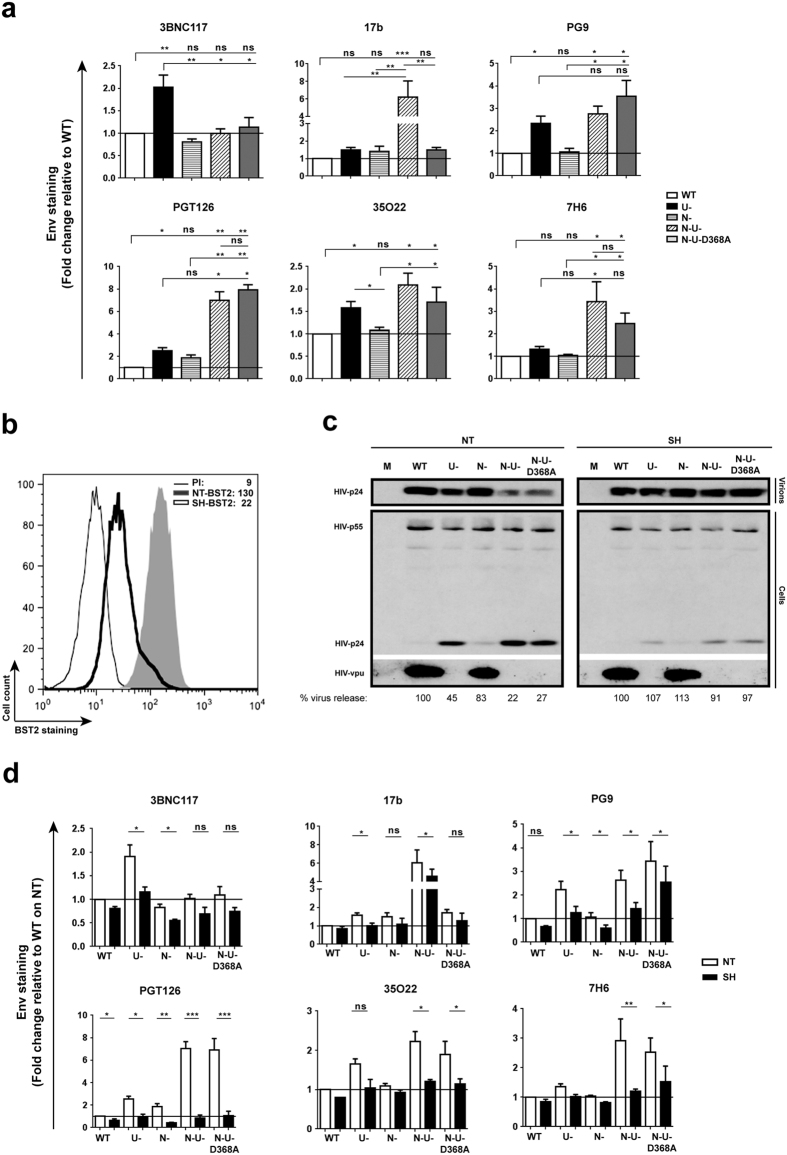
Tethering of HIV virions by BST2 enhances Env recognition by all classes of NAbs. (**a**) Parental CEM CD4^+^ T cells were infected with CCR5-tropic NL4.3.ADA.IRES.GFP WT virus or derivatives lacking Vpu (U-), Nef (N-) or both proteins (N-U-) and examined for Env recognition by NAbs (5 μg/ml for PGT126 and 10 μg/ml for others). The N-U-D368A virus harbours a mutation within the CD4-binding site of Env causing defective CD4-Env engagement. (**b**–**d**) CEM cells expressing (NT) or depleted of (SH) BST2 (**b**) were infected with the viruses as in (**a**) and assessed for HIV particle release by Western blotting (**c**) and for Env recognition as indicated in Panel a (**d**). (**a**) Env staining on GFP^+^ parental CEM T cells. MFI levels on WT-virus infected cells were set at 1 (reference). Shown is a mean fold change of each condition relative to this reference (n = 3 to 4). In this Panel, two-way Anova analysis of variance with Dunn’s *post hoc* tests was used to compare Env recognition levels with different viruses. (**b**) BST2 staining on CEM T cells expressing or depleted of BST2 using anti-BST2 Ab or a rabbit pre-immune (PI) serum as control. Indicated inside the graph are MFI values for GFP^+^ cells from a representative analysis. (**c**) An example of viral particle release with mock (M)-infected cells used as control. Parallel virions and cells were analyzed for total Gag proteins and Vpu. Underneath the immunoblots are quantifications of the densitometry signals. Percentages of virus release were determined as a ratio of virion-associated Gag signals (corresponding to mature p24) over all cell-associated Gag signals (corresponding to p24 and precursor p55) × 100. Virus release by WT virus-infected cells of each cell line (NT or SH) was set at 100. (**d**) Env staining on GFP^+^ cells (n = 3 to 4). MFI levels on BST2-expressing (NT), WT-virus infected cells were set at 1 (reference). Shown is a mean fold change of each condition relative to this reference. In this Panel, paired, two-tailed Student’s *t-*tests were used to compare the effect of BST2 depletion on Env recognition.

First, relative to WT virus, 3BNC117 binding was enhanced by two-fold with ΔU but moderately decreased with ΔN (Fig. 2a, see also Supplementary Fig. S2). The absence of Nef and Vpu did not alter the extent of Env recognition. Although 3BNC117 competes with CD4 for Env binding, the two-fold increase with ΔU virus seemed to argue for a likely contribution from BST2-mediated tethering of virions and not from CD4 abundance since cell-surface CD4 levels were comparable between WT and ΔU virus-infected cells (see Supplementary Fig. S2). Nonetheless, Env recognition with ΔNΔU was not higher than ΔN (or WT), suggesting that perhaps when CD4 accumulated at the surface engages Env, fewer epitopes are available for 3BNC117 binding, despite the increase in Env-containing virions mediated by BST2. Having said that, the fact that Env recognition was not meaningfully increased when comparing ΔNΔU to ΔNΔU D368A was consistent with previous findings showing that binding of CD4-bs Abs such as VRC01 to Env was impaired when there was a mutation at this residue^27^.

Second, while there was a slight increase in 17b binding with ΔU or ΔN virus, the enhancement was highly synergistic with ΔNΔU (~6-fold increase), mirroring our previous finding with CD4-induced A32^19^, and highlighting the importance of CD4 accumulation (see Supplementary Fig. S2) for effectively unmasking the CD4i 17b epitope. However, the fact that the presence of the D368A mutation almost abolished such enhancement differs from what had been observed with A32^19^ and indicates a rather significant necessity of CD4-Env engagement in the exposure of epitope, at least at the level of Env recognition. Interestingly, the overall profile of 7H6 binding was very similar to that of 17b except that the decrease in Env staining with ΔNΔU D368A compared to ΔNΔU appeared statistically insignificant.

Third, for PG9, PGT126 and 35O22 a meaningful increase of 1.5–3 fold in binding across all ΔU viruses was observed, and the presence of the D368A mutation did not negatively affect Env recognition. Further, while there was an increase in Env recognition when comparing ΔNΔU to ΔU for PG9 and PGT126, the difference was significant only for PGT126. At this point, we do not have an explanation for this observation with respect to a role of Nef. Noteworthy, we detected a slight Nef-mediated down-regulation of BST2 on CEM CD4^+^ T cells (see Supplementary Fig. S2). While this effect of Nef on BST2 could have contributed to reduced Env recognition observed with ΔU, the finding might also be an indirect consequence of Nef having an effect on cellular factor(s) that could be involved in the exposition of these epitopes. Overall, these results suggest that differences in activities of Nef and Vpu variants may modulate the extent of Env recognition and, as such, may partially explain the highly variable levels of Env exposure and ADCC observed between isolates (Fig. 1d and e).

Importantly, similar analyses with primary CD4^+^ T cells showed comparable patterns of Env recognition by most antibodies, with two exceptions (see Supplementary Fig. S3). Firstly, a more profound enhancement in Env staining by PG9 and PGT126 was seen with ∆U virus-infected primary cells. It is possible that the lack of Nef-induced down-regulation of BST2 expression on primary cells (see also Supplementary Fig. S3) could lead to more efficient virion tethering by BST2 and thus better Env recognition by PG9 and PGT126. Secondly, unlike in CEM cells (Fig. 2a), PGT126 recognized its epitope on ∆N∆U virus-infected primary cells with significantly less efficiency. At this juncture, it is not clear why there is such a difference between these two cellular models. Despite enhanced recognition in the absence of Vpu-mediated BST2 antagonism, the absence of Nef appears to reduce recognition of PG9 and PGT126 epitopes. Whether this difference is linked to Nef targets SERINC 3 and SERINC 5, which are expressed in primary CD4^+^ T cells but not in CEM cells^28^,^29^ is a possibility that requires further investigation.

### BST2-mediated virion tethering enhances Env recognition and ADCC activity by NAbs

We next asked if BST2 depletion^19^ from CEM CD4^+^ T cells (Fig. 2b) would affect Env recognition and ADCC activities by NAbs through modulation of virion tethering at the cell surface. We first confirmed that BST2 depletion mediated a change in virus particle release. Indeed, when BST2 was expressed (NT), virus release from ∆U virus-infected cells was about 45% of the WT (note the accumulation of p24 in ∆U virus-infected cells compared to WT) (Fig. 2c). A minor effect of Nef on particle release was also observed (compare lanes ∆U to ∆N∆U or WT to ∆N in NT cells), consistent with its slight ability to down-regulate BST2 on infected CEM T cells (see Supplementary Fig. S2). In contrast, when BST2 was depleted (SH), viral particle release was comparable between the viruses, confirming the predominant role of BST2 in virion tethering at the cell surface.

Figure 2d shows how BST2 depletion affects Env recognition by different NAbs. First, binding of 17b to infected cells was evidently decreased in BST2-depleted cells. Although the difference was statistically significant for ∆U and ∆N∆U viruses (*P *< 0.05, paired Student’s *t*-test), the extent of attenuation was not as pronounced as that observed with the other Abs. Indeed, the effect was most striking with PGT126 where Env recognition was not only meaningfully decreased but a difference of as much as 6-fold was also noted for ΔNΔU and ΔNΔU D368A viruses. The fact that Ab binding was reduced with WT and ΔN viruses was not unexpected given the incomplete depletion of BST2 expression in “SH” CEM T cells (Fig. 2b). In consequence, the observation was likely attributed to a loss of BST2 molecules at virion-assembly sites that would have normally been present on BST2-expressing cells during infections with WT or ΔN viruses^17^,^18^. Taken together, the data further highlight the importance of BST2-mediated virion-tethering in Env recognition by NAbs, although the effect of BST2 depletion varied depending on the class.

**Figure 3:**
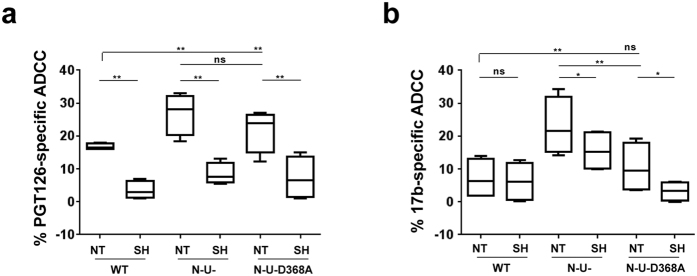
BST2 depletion attenuates ADCC activities mediated by both CD4-induced and CD4-non-induced NAbs. CEM CD4^+^ T cells expressing (NT) or depleted (SH) of BST2 were infected with CCR5-tropic NL4.3.ADA.IRES.GFP WT, N-U- or N-U-D368A viruses as indicated in Fig. 2 legend and examined for ADCC susceptibility mediated by (**a)** CD4-non-induced PGT126 or (**b**) CD4i 17b Abs as described in Fig. 1 legend. Panels a and b summarize results from at least 4 donors. In both Panels, paired, two-tailed Student’s *t-*tests, were used to compare the effect of BST2 depletion on ADCC activity with each virus.

To determine whether such changes in Env binding (Fig. 2d) would accordingly affect ADCC, we assessed susceptibility of CEM CD4^+^ T cells infected with WT, ∆N∆U or ∆N∆U D368A viruses to ADCC mediated by CD4i 17b and, as an example, “CD4 non-induced” PGT126. First, when BST2 was present (NT), T cells infected with the ∆N∆U virus were considerably more susceptible than their WT counterparts: an average of about 2-fold higher for PGT126 (Fig. 3a) and 3-fold for 17b (Fig. 3b). Compared to ∆N∆U virus, the near complete loss of 17b-mediated ADCC observed with ∆N∆U D368A was in line with the data on Env recognition (Fig. 2a). Second, in the context of BST2 depletion, we observed statistically significant reduction in PGT126-mediated ADCC to levels that were comparable across the different viruses (*P *< 0.005, paired Student’s *t*-test). The attenuated killing observed with WT virus in BST2-depleted cells (compare NT to SH of WT; Fig. 3a) was consistent with decreased Env binding (Fig. 2d) and could be attributed to the loss of residual BST2 as discussed above. In contrast, the effect of BST2 depletion on 17b-induced ADCC was observed with ∆N∆U virus-infected cells but not with their WT counterparts (Fig. 3b). The latter finding was expected since it was cell-surface CD4 accumulation that contributed to optimal binding of 17b, and CD4 abundance was meaningfully less on WT virus-infected cells relative to their ∆N∆U virus counterparts (see Supplementary Fig. S2). Interestingly, while ADCC mediated by 17b against ∆N∆U D368A virus-infected cells was statistically comparable to that of their WT counterparts, reduced killing of ∆N∆U D368A virus-infected cells was also observed when BST2 was depleted, suggesting that when CD4-Env engagement is impaired, BST2-mediated virion tethering is likely the sole contributor to ADCC enhancement. It is worthy to mention that BST2 depletion seemed to have a more pronounced effect on ADCC than Env recognition (Fig. 2d). Overall, the data underline the relevance of BST2-mediated virion tethering in ADCC function elicited by both CD4-non-induced and CD4i NAbs.

### IFNα treatment enhances Env recognition and ADCC by bNAbs in a BST2-dependent manner

**Figure 4:**
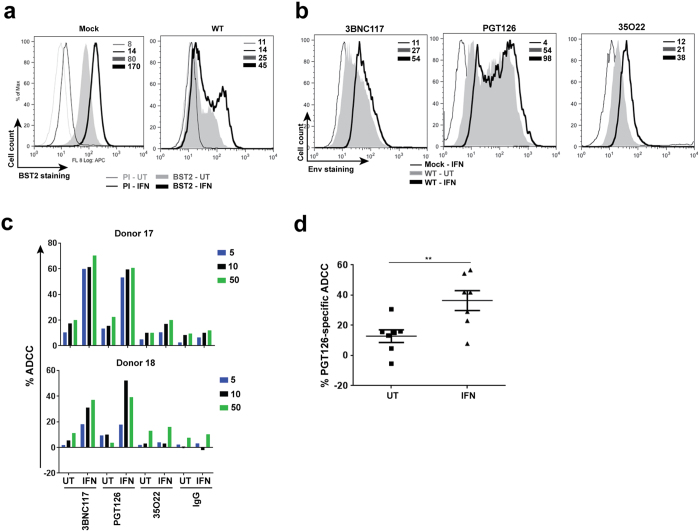
Exogenous IFNα enhances susceptibility of productively infected T cells to ADCC mediated by bNAbs. Parental CEM CD4^+^ T cells were infected or not (mock) with CCR5-tropic NL4.3.ADA.IRES.GFP WT virus for 20 h and then exposed (IFN) or not (UT) to IFNα for about 48 h. Infected cells were analyzed for BST2 or Env expression using (**a**) anti-BST2 or (**b**) the indicated anti-Env Abs, respectively. (**c** and **d)** Cell susceptibility to ADCC was examined as described in Fig. 1 legend. Panel c shows ADCC activities at different concentrations of Abs (in µg/ml) of 2 donors as examples. Panel d summarizes mean target cell killing mediated PGT126 from multiple donors. Each dot represents one PBMC donor. In this Panel, Mann-Whitney *U*-tests were used to compare ranks between UT and IFN-treated groups.

Our findings obtained thus far have demonstrated a close association between virion tethering by BST2 and ADCC enhancement for all classes of NAbs. Given that BST2 is an IFN-I-upregulated restriction factor, we asked if exogenous IFNα would augment ADCC function in a BST2-dependent manner. To this end, we found that IFNα raised BST2 levels by ~2-fold on both mock- and WT HIV-infected T cells. The increase was observed despite the presence of Vpu (Fig. 4a). Further, this upregulation was associated with a ~2-fold enhancement in Env recognition (Fig. 4b) not only by Abs which recognize well the ADA Env like PGT126 and 3BNC117 but also by those that do not such as 35O22 (Fig. 1a). Importantly, in accordance with the IFNα-induced increase in Env binding, target cells became more susceptible to ADCC (Fig. 4c). Given the robustness of PGT126-mediated ADCC response over multiple donors (Fig. 4d), this Ab was chosen for further analyses.

**Figure 5:**
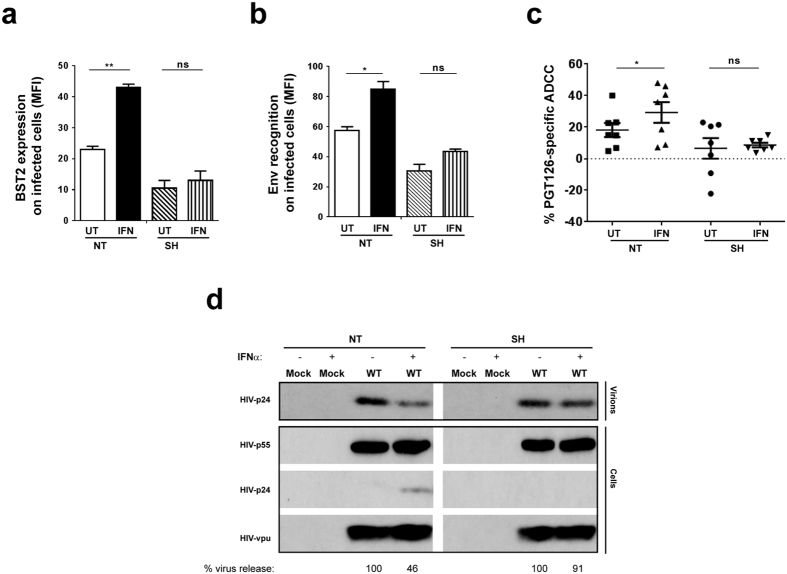
Enhancing physical tethering of HIV virions augments ADCC mediated by bNAbs in a BST2-dependent manner. CEM CD4^+^ T cells expressing (NT) or depleted (SH) of BST2 were infected with CCR5-tropic NL4.3.ADA.IRES.GFP WT virus and treated with IFNα. Infected cells were analyzed for (**a**) BST2 and (**b**) Env expression, (**c**) ADCC susceptibility to PGT126 and (**d**) HIV particle release as described in Fig. 2 legend. Methodology and data analysis are as indicated in the legends for Figs 1 and 2. Each dot in Panel c represents one PBMC donor. Also in Panel c, Mann-Whitney *U*-tests were used to compare ranks between UT and IFN-treated groups for each cell type (NT or SH).

To confirm that such IFNα-mediated augmentation of ADCC was related to enhancement of virion tethering by BST2, CEM CD4^+^ T cells expressing (NT) or depleted (SH) of BST2 (Fig. 2b) were infected with the same CCR5-tropic WT virus as in Fig. 4 and exposed to IFNα. As shown in Fig. 5a, the treatment led to a 2-fold increase in BST2 expression on NT but not on SH cells and correlated accordingly with a statistically significant increase (*P *< 0.005, Mann-Whitney test) in PGT126 binding on BST2-expressing cells but not their BST2-depleted counterparts (Fig. 5b). In consequence, we observed greater ADCC activity in BST2-expressing target cells but not in those depleted of BST2 (Fig. 5c), although the difference between untreated (UT) and IFNα-treated NT cells was less than that for parental CEM CD4^+^ T cells under the same experimental conditions (Fig. 4d). This could be due to inherently higher baseline levels (i.e., IFNα-untreated) of BST2 on uninfected NT cells (Fig. 2b) compared to parental cells (Fig. 4a), which could in turn make the “NT” cells less sensitive to IFNα stimulation than parental cells. Mechanistically, the IFNα-induced increase in ADCC seemed to be due to greater retention of budding virions on infected cells, as evidenced by accumulation of cell-associated p24, and a simultaneous 2-fold decrease in particle release in IFNα-treated, WT virus-infected, BST2-expressing (NT) cells (Fig. 5d). Of importance, this difference was not observed in BST2-depleted CEM CD4^+^ T cells under the same experimental conditions, strongly indicating that IFNα treatment potentiates susceptibility of infected cells to ADCC in a BST2-dependent manner.

### Jurkat-derived T cells latently infected with wild-type HIV are susceptible to ADCC upon reactivation

**Figure 6:**
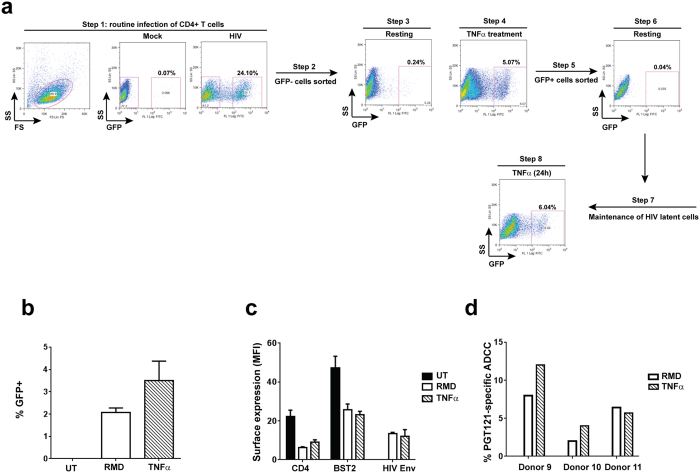
Reactivated latent T cell lines are targets of ADCC. (**a**) Jurkat-derived CD4^+^ T cells were infected with VSV-G pseudotyped, CCR5-tropic NL4.3.ADA.IRES.GFP WT virus and sorted for GFP^-^ cells by flow cytometry. Following a 7-day recovery, GFP^-^ cells were treated with TNFα for 24 h and GFP^+^ cells were sorted and maintained in the absence of TNFα as latent cells. These cells could be reactivated with TNFα or other latency reversing agents. (**b-d**) Resting latent cells from Panel a (Step 7) were exposed to romidepsin (RMD) or TNFα for 9 h and examined by flow cytometry for reappearance of GFP (**b**) and for cell-surface CD4 and BST2 expression, as well as Env recognition by PGT121 (**c**). Reactivated latent cells were assessed for their susceptibility to FACS-based ADCC (**d**). Shown in Panel d are ADCC activities from 3 donors as examples. Methodology and data analysis are as described for Fig. 1.

Having established that productively infected T cells could be eliminated by ADCC, we next investigated whether the same would also be true for reactivated latent T cells. Towards this, a latent cell line, initially infected with VSV-G pseudotyped CCR5-tropic NL4-3.ADA.IRES.GFP WT virus, was established (Fig. 6a). This viral strain was used to prevent potential complications with *de novo* secondary re-infections. A 24-hour treatment with TNFα (2 ng/ml) was routinely used to reactivate up to 10% latent cells. However, since prolonged exposure to a more physiologically relevant latency-reversing agent (LRA) such as romidepsin (RMD)^30^,^31^ was toxic to Jurkat cells, TNFα treatment was kept to 9 h when analyzed with RMD. As expected, HIV latent cells could be reactivated with both LRAs, although TNFα was more effective (Fig. 6b). Importantly, both CD4 and BST2 were down-regulated on reactivated, WT virus-infected latent cells, reflecting *de novo* synthesis of viral proteins such as Nef and Vpu (Fig. 6c). In addition, Env proteins were also expressed at the surface of reactivated cells, as evidenced by PGT121 binding (Fig. 6c). Similar to PGT126, PGT121 binds to V3 base glycans and is sensitive to N332 substitution or removal of the glycan at this residue^32^,^33^. Taken together, these markers indicated that GFP expression was an appropriate marker to measure reactivation. Importantly, reactivated latent cells were susceptible to ADCC regardless of the LRAs used (Fig. 6d). Overall, the data indicate that latently infected T cells could be eliminated via ADCC although their killing remains modest with WT virus.

### Potentiating susceptibility of HIV latent cells to ADCC through enhancement of BST2-mediated virion tethering

Our data thus far have shown that enhancing BST2-mediated virion tethering by IFNα heightened susceptibility of productively infected cells to ADCC (Fig. 5), and that reactivated latent cells were targets of ADCC (Fig. 6). We then asked which conditions would facilitate most efficient elimination of latent cells. In the context of productive HIV infections, we found that target cells infected with the ∆N∆U virus were most susceptible to ADCC compared to those infected with WT or a single mutant virus. Therefore, a T cell line latently infected with a ∆N∆U virus was also established, along with those infected with either ∆N or ∆U virus.

**Figure 7:**
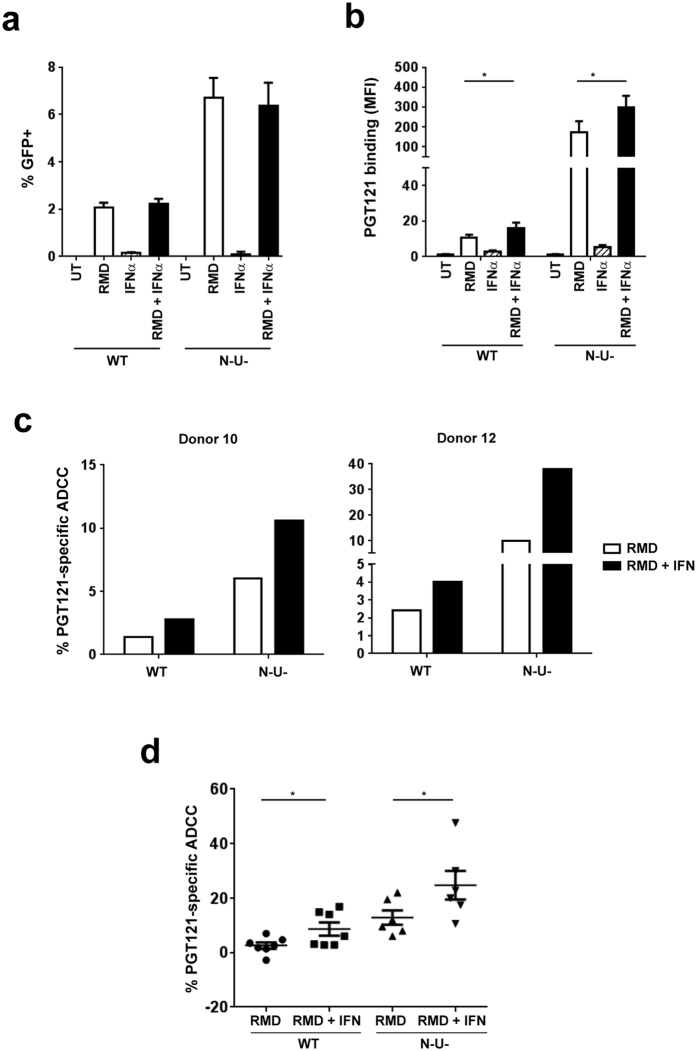
Exogenous IFNα treatment and inactivation of Nef and Vpu enhance ADCC activity against HIV latent cells. Jurkat-based T cells latently infected with CCR5-tropic NL4.3.ADA.IRES.GFP WT or ∆N∆U (N-U-) virus were treated with RMD for 9 h and then with or without IFNα for 15 h. Latent cells were examined by flow cytometry for reappearance of (**a**) GFP, (**b**) Env expression and (**c** and **d**) ADCC susceptibility using PGT121. Panel c indicates ADCC response from 2 donors as examples and Panel d shows averaged Ab-specific ADCC (±s.d.) from 6–7 donors. Each dot represents 1 donor. In Panels b and d, Mann-Whitney *U*-tests were used to compare ranks between RMD and RMD + IFN-treated groups with each virus.

IFNα by itself did not trigger HIV reactivation nor did it augment the effect of RMD (Fig. 7a). Interestingly, virus reactivation by RMD was about 3-fold more efficient in ∆N∆U virus-infected latent cells compared to WT virus. Consistent with findings shown in Fig. 2, Env recognition was markedly pronounced on ∆N∆U virus-infected cells compared to their WT virus counterparts (Fig. 7b). In addition, IFNα treatment led to a 2-fold increase in PGT121 binding on both WT and ∆N∆U virus-infected cells (Fig. 7b) and consequently, a statistically significant (P < 0.05, Mann-Whitney *U*-test) increase in ADCC activity (Fig. 7c and d). Consistent with previous data regarding productive HIV infections, Env recognition and ADCC responses were more pronounced in a latent T cell line carrying the ∆N∆U virus. Taken together, our data clearly indicate that enhancing physical tethering of virions by IFNα treatment and by inactivating BST2 antagonism can accumulatively potentiate latent cells to ADCC induced by broadly NAbs. Indeed, the fact that reactivated latent T cells carrying a ∆U or ∆N∆U virus were comparably more susceptible to PGT121-induced ADCC relative to their WT and ∆N counterparts clearly strengthens this underlying message (see Supplementary Fig. S4).

## Discussion

In this study, we show that NAbs mediate ADCC on T cells infected with different primary virus isolates, although susceptibility of these cells varies with infecting strains. Abs directed against glycans in the V1/V2 apex or V3 base are most efficient at inducing ADCC. Additionally, we provide evidence that Vpu and Nef differentially modulate Env recognition and ADCC activity according to the class of NAbs. Our results highlight that BST2-mediated tethering of nascent virions at the surface of infected cells is instrumental for optimal ADCC function by all classes of NAbs. Indeed, exogenous IFNα treatment potentiates killing of infected cells in a BST2-dependent manner. Importantly, we demonstrate that reactivated latently-infected T cell lines are targets of ADCC although the overall killing remains inefficient. However, as with productively infected cells, inhibiting the ability of HIV to antagonize BST2 and/or IFNα treatment heightened elimination of reactivated cells. Thus, for the first time, we illustrate that this approach could be a new avenue to improve clearance of latent cells.

In our analyses, NAbs that recognize glycan epitopes on V1/V2 or V3 were found to mount a more efficient killing of WT virus-infected T cells compared to those targeting MPER or the gp120-gp41 interface. In many cases, the Abs which effectively blocked HIV entry (IC_50_ ≤ 0.07 μg/ml) were also potent at triggering ADCC, implying their potential relevance in both prophylactic and therapeutic settings of infection. Nevertheless, for some Abs that were less efficient at either or both functions (e.g., 17b and 35O22), the neutralizing capacity was not always predictive of the ADCC potential, and *vice versa*. In fact, findings analogous to ours in this context were recently reported by von Bredow and colleagues^34^. Further, Env recognition and ADCC induced by neutralizing Abs differed among primary isolates but, importantly, these variations were maintained regardless of the frequency of infected cells (*i.e*., % p24^+^) or extent of infection (*i.e*., fluorescence intensity of p24 signal per cell from FACS analyses). In this regard, our data are consistent with recent findings by Bruel and colleagues^24^, suggesting not only heterogeneity at the level of epitopes among viral strains but also potential influence of respective viral proteins on cellular factors that might govern how Env epitopes are displayed at the surface of infected cells. Indeed, functional analyses of Vpu variants from chronic and T/F viruses revealed variations in their ability to down-regulate CD4 and BST2, as well as to enhance virus particle release^35^,^36^,^37^. Additionally, differences in Vpu-mediated targeting of CD4 modulate the amount of sequestered Env and, consequently, incorporation of the glycoprotein into virions^38^. Similarly, patient-derived Nef variants also differ in their ability to down-modulate CD4^39^,^40^, with potential consequences on the levels of Env at the cell surface as well as exposition of CD4i epitopes. Having said that, variations in the percentage of effector cells such as NK cells, as well as their make-up, in different donors could also contribute to variable ADCC responses as clearly shown in this study.

We previously showed that whereas CD4 played a major role in the enhancement of ADCC triggered by the non-neutralizing, CD4i A32 Ab, BST2 had a minor involvement^19^. However, in the context of NAbs as shown here, the contribution of BST2 to ADCC is highly significant. In fact, depletion of BST2 markedly reduces Env binding and ADCC activity mediated by all classes of neutralizing Abs tested. The greatest effect is observed with PGT126. Such a reduction could simply be due to a decrease in Env density otherwise provided by BST2-mediated virion or reduced avidity through fewer cross-linking of epitopes on trapped viral Env spikes to Abs such as PGT126^41^.

In our latency model, a Jurkat-based T cell line infected with the ∆N∆U virus could be reactivated more efficiently than WT virus-infected latent cells, regardless of the type of latency-reversing agent used. Although Nef can boost HIV transcription through NF-κB activation, Vpu seems to be superior at inhibiting this pathway^42^. Thus, it is possible that the absence of Vpu in the ∆N∆U virus provides a cellular environment which enables effective NF-κB-dependent transcription, resulting in better reactivation of latent cells. However, even then, only a small fraction of latent cells could be reactivated at any given time in our model system, echoing the global challenge of reactivating latently infected cells. Although our data show that reactivated latent cells are susceptible to ADCC, the killing remains rather inefficient, especially for those infected with WT or ∆N virus. The fact that the two latent T cell lines carrying the ∆N∆U or ∆U virus were more susceptible to PGT121-mediated ADCC further strengthens the need to employ alternative approaches, such as one that reverts BST2 antagonism by Vpu, that could potentially enhance cell killing. Nonetheless, studies with T cell lines have their own limitations in that they may not necessarily recapitulate what is happening *in vivo*. One such limitation would be the level of Env epitopes expressed at the surface of infected cells. Therefore, further studies are needed to evaluate whether our findings obtained with latent cell lines could be extended to primary cells.

Since ∆N∆U virus-infected T cells are significantly more prone to ADCC than their WT counterparts, the use of small molecules aimed at negating the effects of Vpu and Nef on BST2 and/or CD4 down-regulation would conceivably augment ADCC function. While small molecules such as CD4 mimetics may increase killing of infected cells by only CD4i Abs^23^,^43^, collective data shown here and elsewhere^21^,^22^ demonstrate that enhancing physical tethering of virions by upregulating BST2 expression can improve ADCC functions by both CD4i and CD4-non induced Abs. Indeed, we provide evidence to support that exogenous IFNα sensitizes infected T cells (productive and latent) to ADCC through a process that involves upregulating BST2 expression and enhancing BST2-mediated virion tethering at the surface of infected cells. On this note, our study not only extends previous findings^22^, but also mechanistically links them to virion tethering mediated by BST2. It is worth mentioning that the dose of IFNα used in our study was previously shown to upregulate BST2 to levels comparable to the elevated BST2 levels found in HIV-1 infected patients during the chronic phase of infection^44^. Of additional significance, we identify that ADCC potency of different classes of NAbs could be enhanced using IFNα treatment approach. Lastly, given that these findings together with those reported by others^24^ have indicated a heterogeneous display of Env epitopes at the surface of infected cells, cocktails of ADCC-mediating Abs^12^,^24^ will likely be needed to improve ADCC function.

In summary, our study highlights the role of HIV accessory proteins Nef and especially Vpu in the evasion of ADCC by NAbs. It underscores how physical retention of virions at the cell surface affects susceptibility of infected cells to ADCC, regardless of epitope specificity. The fact that ADCC responses against both productively and latently infected cells could be heightened by enhancing the extent of virion tethering paves the way towards future development of approaches aimed at restoring BST2 restriction in order to facilitate clearance of latent viral reservoirs.

## Methods

### Ethics Statement

This study and experiments were approved by the Research Ethics Review Board of the Institut de Recherches Cliniques de Montréal in accordance with the Declaration of Helsinki. All methods were carried out in accordance with the approved guidelines. Blood samples were obtained from HIV- and HCV- seronegative adults who had given written informed consent.

### Chemicals, antibodies and proviral DNA constructs

Romidepsin (RMD) and phytohaemagglutinin-P were purchased from Sigma Aldrich. Proliferation dye eFlour670 was from Affymetrix. Interferon-α 2a (IFNα) was obtained from PBL Assay Science, tumor necrosis factor-alpha (TNFα) was from BioLegend and human recombinant interleukin-2 (IL-2)^45^ was available through the NIH AIDS Research and Reference Reagent Program. Luciferase Assay System including lysis buffer were from Promega. Rabbit anti-Vpu and pre-immune sera were generated as previously described^46^. Mouse anti-p24 mAb (Cat #: HB9725) was isolated from culture supernatant of hybridoma cells from the American Type Culture Collection (ATCC). PE-conjugated anti-p24 Ab (KC57-RD1) was obtained from Beckman Coulter. PerCP-Cy 5.5-conjugated anti-human CD4 antibody was from BioLegend. Rabbit anti-BST2 Ab was described elsewhere^46^. Purified human serum IgG (Cat #: I4506) was obtained from Sigma Aldrich. AF633-conjugated anti-rabbit IgG and AF647-conjugated anti-human IgG secondary Abs were from Life Technologies.

Broadly neutralizing antibodies including PG9 (IgG1)^47^, PGT121^32^,^41^ and PGT126^32^ were obtained through the NIH AIDS Research and Reference Reagent Program. In addition, 3BNC117 (IgG1) and 10-1074 (IgG1) were from Dr. Michel C. Nussenzweig^48^; 8ANC195 (IgG1) was from Dr. Pamela Bjorkman^49^; 35O22, 7H6 and 10E8 (all were IgG1) were from Drs. Jinghe Huang and Mark Connors^6^,^50^; and 17b (IgG1) was from Dr. James. E. Robinson^51^,^52^,^53^,^54^,^55^,^56^.

Proviral construct CCR5-tropic NL4.3.ADA.IRES.GFP wild-type (WT), which encodes all accessory proteins, and its isogenic derivatives: Vpu-deficient (U- or ΔU), Nef-deficient (N- or ΔN), Nef- and Vpu-deficient (N-U- or ΔNΔU) and ΔNΔU D368A (N-U-D368A) were generated using standard molecular biology techniques as described^19^. ΔNΔU D368A construct contains a mutation within the CD4-binding site of Env at position 368, resulting in impaired CD4-Env interactions.

Infectious molecular clones of transmitted/founder (T/F) viruses: p.WITO.c/2474 (Cat #: 11739), p.CHO77.t/2627 (Cat #: 11742) and p.THRO.c/2626 (Cat #: 11745) were obtained through the NIH AIDS Research and Reference Reagent Program from Drs. John Kappes and Ochsenbauer^57^,^58^,^59^,^60^,^61^.

### Primary cells, T cell lines and infection of T cells

Peripheral blood mononuclear cells (PBMCs) were prepared from whole blood of healthy adults using Ficoll-Hypaque Plus (GE Healthcare). PBMC were cultured overnight in complete RPMI-1640 media [10% fetal bovine serum (FBS) supplemented with L-Glutamine, Penicillin-streptomycin] and 100 U/mL IL-2. Primary CD4^+^ T cells, isolated by negative selection using the human CD4^+^ T cell isolation kit from Miltenyi Biotec, were activated with 5 μg/ml phytohaemagglutinin-P for 48 h and cultured in the presence of 100 U/mL IL-2.

HEK 293 T cells were obtained from ATCC and maintained in complete DMEM media (10% FBS supplemented with Penicillin-streptomycin). Hela TZM-bl and all other T cell lines: CEM.CCR5 CD4^+^, JLTRG-R5^62^ and 1G5 were obtained from the NIH AIDS Research and Reference Reagent Program. Whereas Hela TZM-bl were cultured in complete DMEM media, T cell lines were in complete RPMI-1640 media. CEM.CCR5 CD4^+^ T cells were depleted of BST2 using lentiviral vector particles containing shRNA targeting BST2 (referred to as “SH” cells in text and figures) or control shRNA (referred to as “NT” cells in text and figures) as previously described^19^.

Activated primary CD4^+^ T cells or CEM.CCR5 CD4^+^ T cells were infected, as appropriate, with VSV-G pseudotyped CCR5-tropic NL4.3.ADA.IRES.GFP viruses or T/F viruses to have about 15–30% infected cells at 48 h post infection (for CEM T cells). Primary CD4^+^ T cells were analyzed at 72 h post infection and the infection was in the range of 2–10% GFP^+^ cells depending on donors. In certain experiments, T cells were infected with WT virus for 15 to 20 h and then treated with IFNα (1,000 U/mL) for about 48 h before harvesting for analysis.

### Production of VSV-G-pseudotyped lentiviral vectors and HIV-1 viruses

For virus production, HEK 293 T cells were co-transfected with an HIV proviral construct and pSVCMV-VSV-G using calcium phosphate precipitation method and harvested at 48 h post-transfection^19^. Titration was done by flow cytometry-based analysis of GFP expression in parental CEM.CCR5 CD4^+^ T cells (referred to as CEM in text and legends) for NL4.3.ADA-based viruses or in JLTRG5 for T/F viruses.

### Generation of HIV latent T cells and their reactivation with latency-reversing agents

HIV latent T cell lines were established using a protocol similar to that described by Jordan and colleagues^63^. Briefly, Jurkat-based 1G5 T cells were infected with VSV-G pseudotyped, CCR5-tropic NL4.3.ADA.IRES.GFP WT or its derivatives ∆N (N-), ∆U (U-) and ∆N∆U (N-U-) virus for 4 to 5 days to have about 20–40% infection. GFP^-^ population, which contained uninfected or latently infected cells, were sorted by flow cytometry (purity was approximately 99%). After 7 days of resting, GFP- cells were treated for 24 h with 2 ng/ml TNFα. GFP^+^ cells (about 5–15%) were again sorted and rested in the absence of TNFα, which returned them to quiescent state. Latency status was confirmed by analyzing surface expression of HIV Env, CD4 and BST2 on GFP^+^ cells.

To reactivate the virus, latent 1G5 cells were routinely treated with RMD (5 nM) or as a control, TNFα (2 ng/mL) for 9 h. Twenty-four hours later, cells were analyzed for GFP expression, Env recognition as well as ADCC susceptibility. For experiments involving IFNα treatment, after the 9-hour treatment with LRAs, cells were washed, fresh media containing IFNα (1,000 U/mL) were added and cells were analyzed at 24 h post LRA treatment.

### Flow cytometry

CD4 staining was done as per manufacturer’s protocols. BST2 staining was done as described using anti-rabbit BST2 Ab or as a control, a rabbit pre-immune (PI) serum^19^. For Env staining, at 2 days post-infection, cells were stained with human anti-Env Abs (1–50 μg/ml) for 45 min at 4 °C. Fluorescence signals were revealed using an AF647-conjugated anti-human Ab. Mock-infected cells were used as control. Cells were analyzed on a CyAn ADP analyzer. Voltage settings on the CyAn were set as appropriate when analyzing samples from experiments that included mutant viruses (versus those with WT virus alone) to allow for appropriate capture of fluorescence signals.

For intracellular p24 staining, T cell cultures were fixed and permeabilized using the BD Cytofix/Cytoperm kit (Cat #554714) as per manufacturer’s instructions. Following a 15-min incubation at 4 °C with a blocking buffer (1X BD Perm/Wash buffer containing 0.5 mg/ml rabbit IgG and 5% FBS), cells were stained with PE-conjugated anti-p24 antibody for 10 min at 4 °C and then washed 2 times with BD Perm/Wash buffer (1X). Mock-infected cells were used as control. Cells were analyzed on a CyAn ADP analyzer.

### Neutralization assay

Two to five-fold serial dilutions of Abs (50 μl) were incubated in a 96-well plate with CCR5-tropic NL4.3.ADA.IRES.GFP WT virus (50 μl) for 90 min at 37 °C. Hela TZM-bl cells, which express an HIV LTR-driven luciferase gene, were added to the Ab and virus mixture at 10,000 cells in 100 μl. Assays were done in three replicates except for negative (cells alone) and positive (cells with virus but no Ab) controls which had five replicates. At 48 h post infection, supernatant was removed and cells were lysed using 150 μl 1 × luciferase lysis buffer. Clarified cell lysates were read on a luminometer for luciferase activity (relative light units, RLUs). Neutralization efficacy was calculated as: 100 × [(RLUs positive control − RLUs test sample)/(RLUs positive control − RLUs negative control)]. Data were analyzed using Prism and IC_50_ values were calculated by the software using Log inhibitor *vs.* Response (Variable slope) equation. IC_50_ (μg/ml) was the concentration at which 50% of infection was blocked.

### Antibody-dependent cell-mediated cytotoxicity assay (ADCC)

The protocol is a modified version of that published previously^19^. For bioluminescence-based assay by luciferase, 50,000 CEM CD4^+^ target (T) cells plated in a 96-well V-bottom plate were exposed to NAbs (up to 50 μg/ml) or human control IgG Ab for 30 min at room temperature and then incubated with PBMCs (effectors) at a ratio of 10 effectors per 1 target for 6 h at 37 °C. For each condition, the assay was done in biological duplicates or triplicates. Subsequently, cells were spun at 400 × *g*, supernatant completely removed and cell pellet lysed for luciferase expression as described for “Neutralization assay”. Clarified cell lysates were read on a luminometer for luciferase activity (RLUs). Percent of cell lysis was determined as indicated below. For FACS-based ADCC assay with latent cells as targets, effector cells were labelled with eFlour670 proliferation dye and incubated with target cells at E:T ratios of 0.25 to 5. After 4 h of incubation, cells were collected by centrifugation, fixed in 1% paraformaldehyde and analyzed for GFP expression. Percentage of Ab-specific ADCC was determined as 100 × [(% GFP^+^ or RLUs)_effectors + control Ab _− (% GFP^+^ or RLUs)_effectors + test Ab_]/[(% GFP^+^ or RLUs)_effectors + control Ab_]. In certain experiments where cell killing mediated in the absence or presence of (control or test) antibody needs to be separately conveyed, the following formula is used to determine percentage of cell lysis: 100 × [(% GFP^+^ or RLUs)_no effectors _− (% GFP^+^ or RLUs)_effectors + test agent_]/[(% GFP^+^ or RLUs)_no effectors_]. “Test agent” in the second formula could be in the absence of Ab (to yield antibody-unrelated killing), or presence of Ab (to yield standard ADCC). Standard ADCC by test Ab must be subtracted from that of control IgG to obtain Ab-specific ADCC.

### Viral particle release assay

CEM CD4^+^ T cells were infected for 48 h with CCR5-tropic NL4-3.ADA. IRES.GFP WT strain or its derivatives deficient of Nef and/or Vpu. In certain experiments, cells were treated with IFNα (1,000 U/mL) at 15–20 h post infection and viral particle release assays were performed at 48 h post infection as described before^19^. Western blotting was performed using Abs specific for Vpu or Gag.

### Statistical analyses

Data were analyzed using GraphPad Prism. Descriptive measures (mean, median, min/max range and percent) were used to summarize the data. Unless otherwise stated, data were expressed as average ± sem. Two-way Anova analysis of variance with Dunn’s *post hoc* tests was used to compare levels of Env recognition by different Abs on T cells infected with different viruses. Paired, two-tailed Student’s *t-*tests, were used to compare the effect of BST2 depletion on Env recognition. Two-tailed Mann-Whitney *U*-tests were used to compare ranks between two treatment groups. P values of ≤ 0.05 were considered statistically significant: * ≤ 0.05; ** ≤ 0.005; *** ≤ 0.0005; and ns, not significant.

## Additional Information

**How to cite this article**: Pham, T. N. Q. *et al.* Enhancing Virion Tethering by BST2 Sensitizes Productively and Latently HIV-infected T cells to ADCC Mediated by Broadly Neutralizing Antibodies. *Sci. Rep.* **6**, 37225; doi: 10.1038/srep37225 (2016).

**Publisher’s note**: Springer Nature remains neutral with regard to jurisdictional claims in published maps and institutional affiliations.

## Supplementary Material

Supplementary Information
