# Supplementary material for: Enhancing Virion Tethering by BST2 Sensitizes Productively and Latently HIV-infected T cells to ADCC Mediated by Broadly Neutralizing Antibodies
Source: Sci Rep. 2016 Nov 17;6:37225. doi: 10.1038/srep37225 (PMC5112552; doi:10.1038/srep37225)
Supplement: Supplementary Information [file srep37225-s1.pdf]

Enhancing Virion Tethering of HIV by BST2 Sensitizes Productively and Latently Infected T cells to ADCC Mediated by Broadly Neutralizing Antibodies

Tram N.Q. Pham[1], Sabelo Lukhele[1], Frédéric Dallaire[1], Gabrielle Perron[1]

Éric A. Cohen[1,2*]

SUPPLEMENTARY INFORMATION

Supplementary Figure Legends

Fig. S1. Neutralization potency and ADCC potential of anti-HIV antibodies. CEM $CD4^+$ T cells were infected with prototypic virus CCR5-tropic NL4.3.ADA.IRES.GFP WT strain and at 2 dpi infected cells were examined for (**a**) cell-surface Env expression and (**b**) susceptibility to ADCC using different concentrations of NAbs (in µg/ml). (**c**) Neutralizing antibodies were evaluated for their ability to neutralize cell-free virus. Extent of Env staining on GFP^+ T cells over a range of concentrations was expressed as averaged median fluorescence intensity (MFI) units from 3 to 4 experiments. PBMCs from healthy donors were used as effector cells. Percentages of Ab-specific ADCC were determined following subtraction of cell lysis mediated by control IgG from that by test antibody. Mean ADCC activity from at least 4-7 donors is indicated. Serial dilutions of known concentrations of NAbs (or as control, PBS) were pre-incubated with prototypic CCR5-tropic NL4.3.ADA.IRES.GFP WT virus for 45 min at 37 ^oC. Luciferase-expressing HeLa TZMbl reporter cells were subsequently added to Ab-virus complexes. Forty-eight hours later, infected cells were lysed and relative light units enumerated on a luminometer. Data were analyzed using GraphPad Prism. Shown are mean IC_{50} from 2 to 3 experiments; ND, not done.

Fig. S2. Cell-surface envelope, CD4 and BST2 expression profiles on HIV-infected CEM $CD4^+$ T cells. Parental CEM $CD4^+$ T cells were infected or mock-infected with CCR5-tropic NL4.3.ADA.IRES.GFP WT virus or derivatives lacking Vpu (U-), Nef (N-) or both (N-U-). The N-U-D368A virus has a mutation within the CD4-binding site of Env

causing defective CD4-Env interactions. At 2 dpi, infected cells were analyzed by flow cytometry for (**a**) Env using indicated NAbs and (**b**) CD4 using anti-CD4 Ab. (**c**) Alternatively, CEM $CD4^+$ T cells expressing (NT) or depleted (SH) of BST2 were infected with the indicated viruses as in Panel a and analyzed for cell-surface BST2 using anti-BST2 Ab or pre-immune serum (PI) as control. Shown in Panels a and c are expression levels on GFP^+ cells presented as MFI units from a representative experiment. Panel b indicates mean CD4 expression from 3 experiments.

Fig. S3: Cell-surface envelope and BST2 expression profiles on HIV-infected primary $CD4^+$ T cells. Activated $CD4^+$ T cells were infected or mock-infected with CCR5-tropic NL4.3.ADA.IRES.GFP WT virus or derivatives lacking Vpu (U-), Nef (N-) or both (N-U-). The N-U-D368A virus has a mutation within the CD4-binding site of Env causing defective CD4-Env interactions. At 3 dpi, infected GFP^+ cells were analyzed by flow cytometry for (**a**) Env using indicated NAbs or (**b**) BST2 using anti-BST2 Ab. Shown in both Panels a and b are mean expression levels on GFP^+ cells presented as MFI units from 3 different donors.

Fig. S4: Reactivated latent T cell lines are targets of ADCC and Vpu dampens PGT121-mediated ADCC response. Jurkat-based T cells latently infected with CCR5-tropic NL4.3.ADA.IRES.GFP WT or its mutant derivatives lacking Vpu (U-), Nef (N-) or both (N-U-) viruses were treated with TNFα and examined for their susceptibility to ADCC mediated by PGT121. PBMCs from healthy donors were used as effector cells.

(**a**) Examples of ADCC response from 2 donors. (**b**) Compilation of PGT121-specific ADCC. Boxes and whiskers graph shows median ADCC from 4 donors.

a

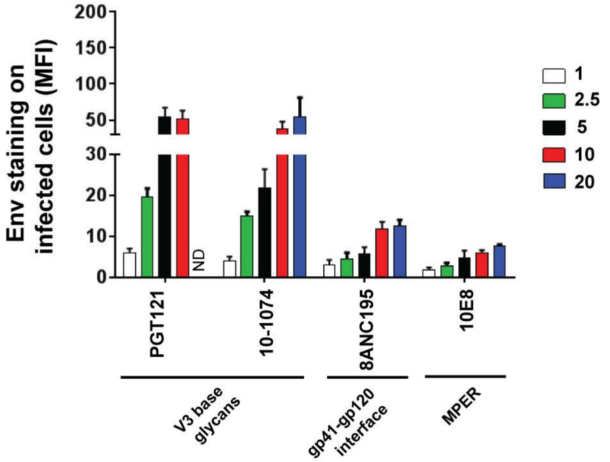

b

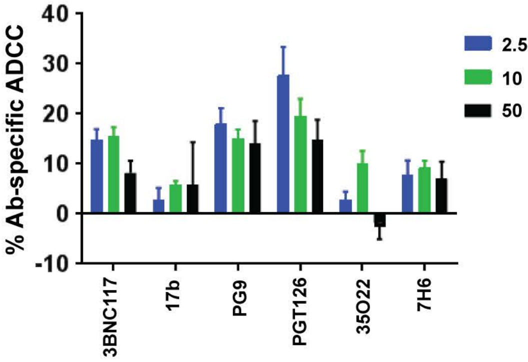

c

Antibody	Epitope (Env region)	IC_{50} ± S.D. (µg/ml)
3BNC117	CD4-bs (gp120)	0.07 ± 0.03
17b	CoR-bs (gp120)	1.30 ± 2.08
PG9	V1/V2 apex (gp120)	0.07 ± 0.02
PGT121	V3 base glycans (gp120)	0.04 ± 0.02
PGT126	V3 base glycans (gp120)	0.04 ± 0.01
35O22	gp41-gp120 interface	17.78 ± 9.50
7H6	MPER (gp41)	0.26 ± 0.10

Fig. S1

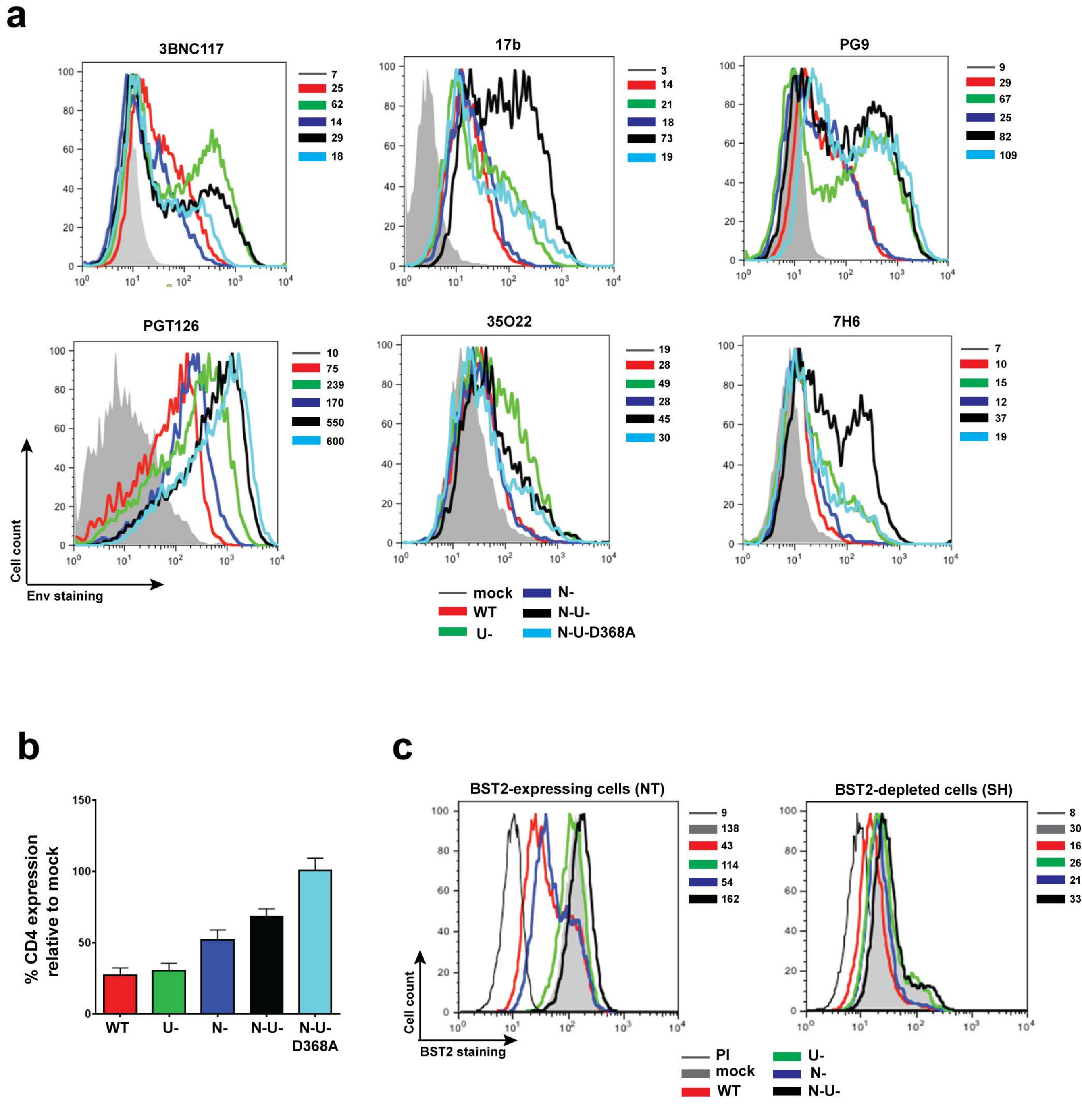
a
3BNC117
7
25
62
14
29
18
17b
3
14
21
18
73
19
PG9
9
29
67
25
82
109
PGT126
10
75
239
170
550
600
35O22
19
28
49
28
45
30
7H6
7
10
15
12
37
19
Cell count
Env staining
mock
WT
U-
N-
N-U-
N-U-D368A
b
% CD4 expression relative to mock
WT
U-
N-
N-U-
N-U-D368A
c
BST2-expressing cells (NT)
9
138
43
114
54
162
BST2-depleted cells (SH)
8
30
16
26
21
33
Cell count
BST2 staining
PI
mock
WT
U-
N-
N-U-

Fig. S2

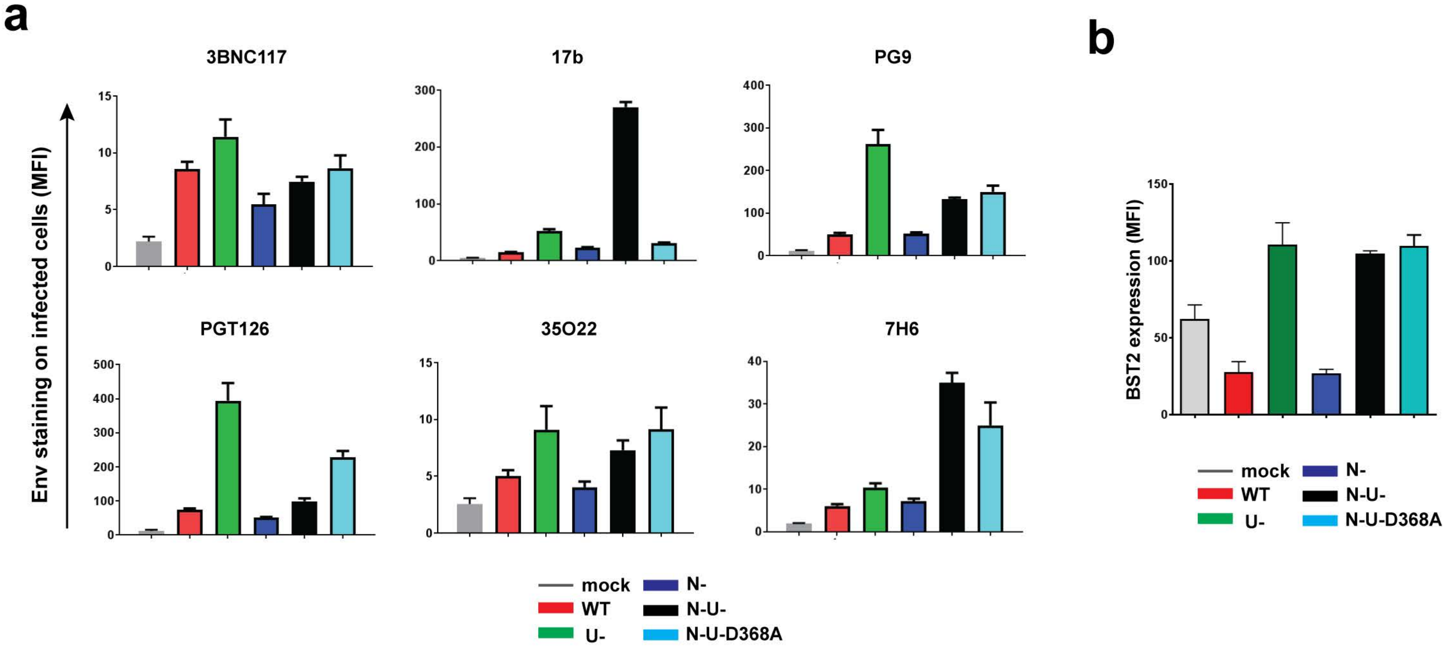
a
3BNC117
17b
PG9
PGT126
35O22
7H6
Env staining on infected cells (MFI)
mock
WT
U-
N-
N-U-
N-U-D368A
b
BST2 expression (MFI)
mock
WT
U-
N-
N-U-
N-U-D368A

Fig. S3

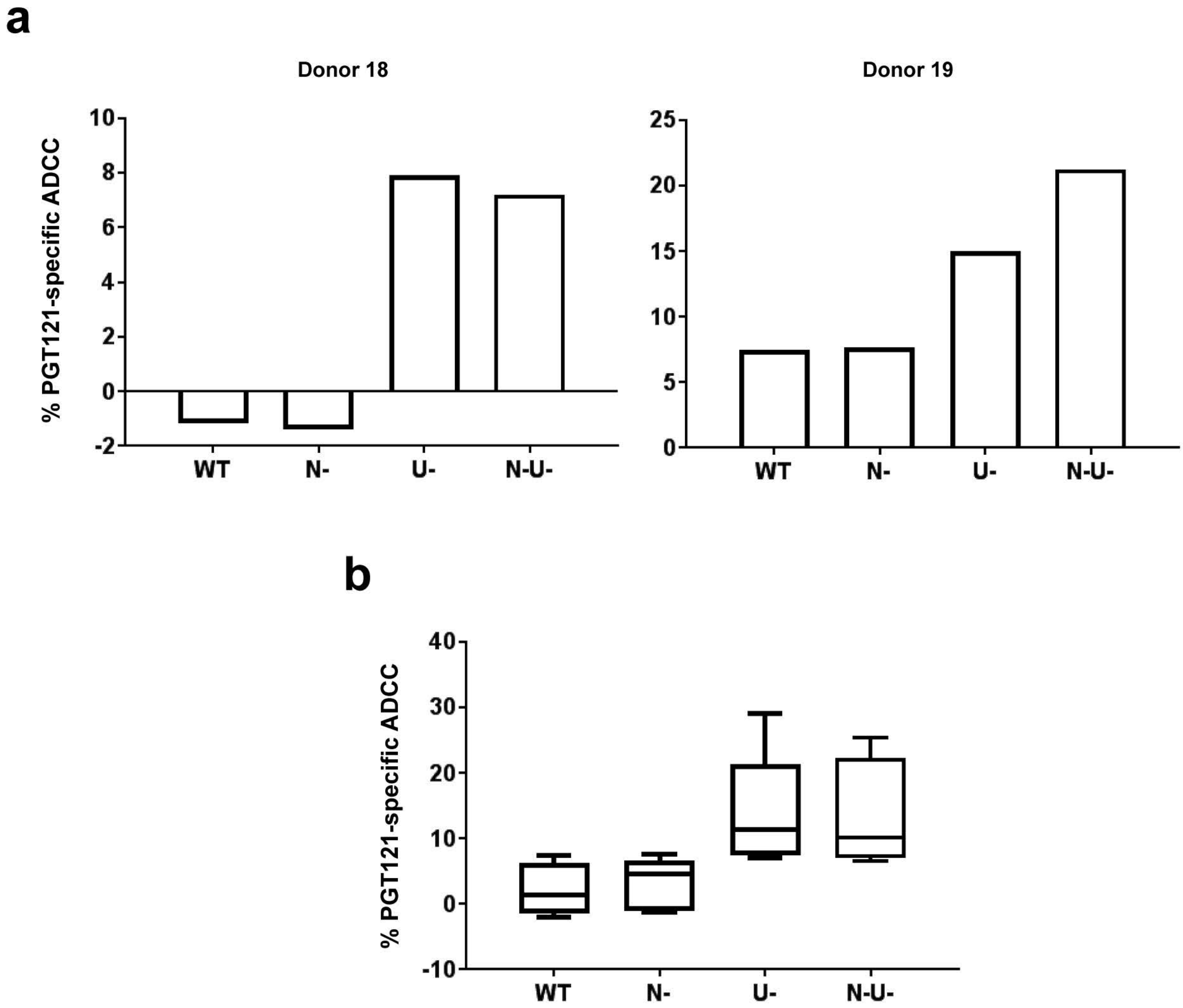
a
Donor 18
Donor 19
% PGT121-specific ADCC
10
8
6
4
2
0
-2
25
20
15
10
5
0
WT
N-
U-
N-U-
b
40
30
20
10
0
-10

Fig. S4
